# A Camera Calibration Method for Temperature Measurements of Incandescent Objects Based on Quantum Efficiency Estimation

**DOI:** 10.3390/s25103094

**Published:** 2025-05-14

**Authors:** Vittorio Sala, Ambra Vandone, Michele Banfi, Federico Mazzucato, Stefano Baraldo, Anna Valente

**Affiliations:** Automation, Robotics and Machines, Institute of Systems and Technology for Sustainable Production, Department of Innovative Technologies, University of Applied Science and Arts of Southern Switzerland, via La Santa 1, 6962 Lugano, Switzerland; ambra.vandone@supsi.ch (A.V.); michele.banfi@supsi.ch (M.B.); federico.mazzucato@supsi.ch (F.M.); stefano.baraldo@supsi.ch (S.B.); anna.valente@supsi.ch (A.V.)

**Keywords:** thermal imaging, camera quantum efficiency, multispectral camera

## Abstract

**Highlights:**

**What are the main findings?**

- We present a novel method for calibrating the temperature-to-color relation (Planckian locus), enabling the measurement of incandescent object temperatures using a standard Bayer-pattern CMOS camera.
- Our method estimates the quantum efficiency of the entire optical system—including the camera, lens, and filters—using images of a black body furnace.

**What is the implication of the main finding?**

- Our method enables high-resolution, high-frame-rate temperature measurement of glowing hot materials in the range of 700–2500 °C with standard cameras and lenses improving process control in metal, semiconductor, and ceramic industries, and accelerating the development of additive manufacturing.
- Our technique is designed for standard cameras and lenses, potentially enhancing environmental monitoring by estimating the temperature in wildfires and volcanic activity, improving predictive modeling and hazard assessment.

**Abstract:**

High-temperature thermal images enable monitoring and controlling processes in metal, semiconductors, and ceramic manufacturing but also monitor activities of volcanoes or contrasting wildfires. Infrared thermal cameras require knowledge of the emissivity coefficient, while multispectral pyrometers provide fast and accurate temperature measurements with limited spatial resolution. Bayer-pattern cameras offer a compromise by capturing multiple spectral bands with high spatial resolution. However, temperature estimation from color remains challenging due to spectral overlaps among the color filters in the Bayer pattern, and a widely accepted calibration method is still missing. In this paper, the quantum efficiency of an imaging system including the camera sensor, lens, and filters is inferred from a sequence of images acquired by looking at a black body source between 700 °C and 1100 °C. The physical model of the camera, based on the Planck law and the optimized quantum efficiency, allows the calculation of the Planckian locus in the color space of the camera. A regression neural network, trained on a synthetic dataset representing the Planckian locus, predicts temperature pixel by pixel in the 700 °C to 3500 °C range from live images. Experiments done with a color camera, a multispectral camera, and a furnace for heat treatment of metals as ground truth show that our calibration procedure leads to temperature prediction with accuracy and precision of a few tens of Celsius degrees in the calibration temperature range. Tests on a temperature-calibrated halogen bulb prove good generalization capability to a wider temperature range while being robust to noise.

## 1. Introduction

Spatially resolved thermal measurements [1] of incandescent objects [2] have relevant applications in several fields ranging from the industrial production of metals [2,3] and ceramics [4] to monitoring the eruptive activities of volcanos [5,6]. Recent attention on the monitoring and controlling of incandescent or molten metals is being triggered by the rapid development of additive manufacturing deposition processes [7,8]. In such techniques, a spatially focused energy source like a laser, an electron beam, or an electric arc determines the local fusion of a metallic substrate [8,9] generating a melt pool. The melt pool is enlarged by adding extra material, provided by a wire, or ejected as powder by nozzles that solidifies, depositing a solid track when the heating source moves away. The part to be printed is sliced into multiple tracks in several layers, defining a sequence of instructions that are executed by the printing machine [9]. Although a lot of research efforts have been done [10] some issues are still open: the geometrical match of the part with the design cannot be guaranteed for all geometries due to excessive or reduced metal deposition [11]. Both defects could also be related to the spatial temperature distribution in the part that influences the melt pool temperature. A warmer melt pool will solidify later, collecting more raw material and potentially deforming the part, thus affecting the final geometry [11]. High-speed thermal monitoring, based on pyrometers [12,13] or mid-infrared cameras [14] proved to be able to control and stabilize the deposition rate [10] and to monitor porosity [14]; nonetheless, the pyrometer-based control suffers significant limitations in complex paths [15]. Pyrometers lack spatial resolution that is important to monitor the geometry of the track [16,17]. Temperature measurements by mid-infrared cameras are subject to the uncertainty of the emissivity coefficient, which changes in the phase transitions or due to oxidation [13,18]. To overcome this limitation, the ratio pyrometers split light into two spatially superimposed narrow wavebands, reducing the effect of uncertainty on emissivity on temperature estimation [12,13,15]. Ratio pyrometers provide accurate temperature measurements but lack spatial resolution [13]. Implementing the technique of ratio pyrometry with cameras requires two filtered narrow bands and spatially superimposed images [13], which requires a dedicated optical setup with high cost and complexity [19]. A wide application of additive manufacturing techniques requires reducing the cost of the monitoring systems while improving speed and spatial resolution, which could be obtained by substituting the existing systems, mid-infrared cameras or the ratio pyrometer with the much less expensive and commonly diffused visible CMOS cameras based on the Bayer pattern filter [17,20,21].

### 1.1. Temperature Measurement with Cameras

Early research on incandescent object temperature measurement relies on monochrome cameras [22,23,24], proposing calibration models directly relating the gray level to temperature. To improve robustness against emissivity coefficient variation, systems based on the ratio pyrometry principle [13], also known as two-color, have been developed. The ratio pyrometry algorithm requires obtaining two spatially superimposed images representing the same scene at the same time in two different spectral bands, obtained by filtering incoming light with two narrow band spectral filters [19,25,26,27,28,29]. All these systems require a specific optical setup to guarantee the spatial superposition of the images acquired at the different wavebands: a dichroic mirror [25,26,28] and a filter assembly [27] or a plenoptic lens [19,29]. Using a color or a multispectral camera with color filters positioned on each pixel of the CMOS sensor avoids the need for a dedicated optical setup. Nonetheless, the filters installed on the sensor present a broad spectral transmission making the ratio pyrometry algorithm not fully applicable [13,17]. Several calibration approaches have been proposed specifically to address the challenge posed by the broad spectral response of the sensor’s filters, which differs from the idealized narrow-band filter assumption [17,20,30,31]. One of the proposed calibration approaches maps the color space of the camera into the xy color space defined by the CIE 1931 standard [32] with a conversion matrix and then rely on the standard observer to model the Planckian locus that associates the chromaticity values to temperature. The approach of converting color space is tested only above 1700 K [21]. Nonetheless, this method appears not ready for industrial application since a robust calibration method is missing. The conversion to xy observer is sensitive to white balance setting and to the color conversion matrix optimization. Both of these are strongly illumination-dependent, requiring specific lighting equipment for reliable calibration. A recent approach models the spectral overlap of RGB cameras by mapping the red, green, and blue channels with equivalent wavelengths to rely on the two-color method [17]. In addition, the blue channel is subtracted from the others to compensate for the near-infrared light collected by the camera due to the leaks of the filters, but this approximation holds only up to around 2000 K. Processing of high melting point metals in additive manufacturing easily overcomes this limit [33,34], requiring the development of an algorithm that better approximates the physical modelling of the system and generalizes to higher temperature. A preliminary test has been performed with multispectral cameras with narrow band filters by optimizing a matrix to avoid band crosstalk. The optimization is specific for the optical system [34]. Calibration data are collected by looking at a black body furnace at a known temperature [34,35]. The anti-crosstalk matrix appears not applicable to the RGB cameras due to the broader spectral bands, requiring a full description of the physics of the system. A full model regressing temperature from the RGB color space is proposed in [36] but the method proposed is limited to the working range of the black body used for calibration, typically too low for additive manufacturing.

### 1.2. Planckian Locus in RGB Color Space

To overcome the limitations related to limited temperature range of the black body source used for calibration while keeping into account the complexity of the system, a consistent physical model has to be developed. Therefore, the Planckian locus, relating chromaticity to temperature, is obtained by integrating the radiated emission by incandescent objects at a given temperature in the wavelength range of the camera. Radiated light intensity is determined by the Planck’s law, modeling the incandescent object as a graybody with a constant emissivity coefficient. Radiated light is attenuated by the transmission of the optical chain: including lens, bandpass or attenuation filters, and filters installed directly on the sensor. The latter are modeled with the quantum efficiency parameter of the camera [17,31,36]. Therefore, estimating the transformation from observed colors to temperature requires an accurate knowledge of the quantum efficiency (QE) of the camera together with its optical chain since uncertainty on QE derives also from variability among sensors, filters, and lenses that should be nominally identical, being the same model [37]. QE uncertainty could affect the calibration, requiring compensation to run the model [17].

### 1.3. Quantum Efficiency

The QE of the cameras [37] is measured according to the EMVA 1288 standard [38]: monochromatic light of known intensity is conveyed on the sensor by a monochromator, scanning the wavelength. The intensity of the signal generated by the camera is collected for each wavelength and each band. The procedure requires specific instrumentation, dedicated labs, and optical expertise [17,30]. Alternative methods infer QE from several light-emitting diodes with different peak emission wavelengths [39] or sets of filters [40] or infer it with deep learning networks [41]. These methods require dedicated tools and provide limited precision. Therefore, a method specifically dedicated to optimizing QE for temperature calibration that is strongly sensitive to the near-infrared region of the spectrum between 700 and 1000 nm is not available.

### 1.4. Regression of Chromaticity to Temperature

Once the Planckian locus is determined, the temperature needs to be regressed from chromaticity. The proposed methods are approximating the CIE 1931 Planckian locus with a function [42,43], interpolation [44], matrix search [36] or regressing with a neural network [21]. Function approximation is defined only in limited temperature ranges on the CIE 1931 Planckian locus; therefore, it cannot be generalized to a generic locus. The neural network shows higher robustness on noisy data than other methods [21], allowing generalization on multispectral cameras not supported by interpolation [44] or matrix search [36].

### 1.5. Our Approach

In this paper, we present a novel method for temperature measurement in industrial process monitoring of molten metals, with particular emphasis on applications in additive manufacturing. The approach is designed to operate over the broad temperature ranges typical of arc discharge and laser-based processes, while supporting acquisition rates of tens of Hz and spatial resolutions down to tens of micrometers. Unlike state-of-the-art techniques that prioritize accuracy within narrow calibration ranges or rely on dedicated optical setups—such as two-color pyrometry methods, which require spatially superimposed images filtered through narrow spectral bands—our method emphasizes robustness and generalizability. Specifically, our method enables temperature estimation using widely available and relatively low-cost color or multispectral cameras.

**Figure 1:**
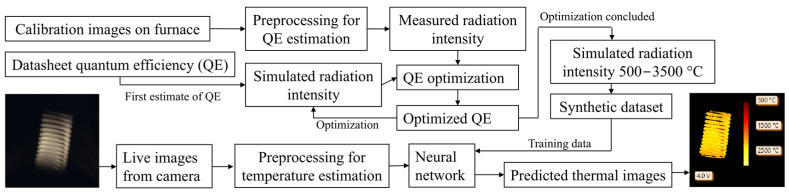
Scheme of the steps for camera calibration and temperature estimation.

To address the limitations posed by the complex, wavelength-dependent quantum efficiency of such sensors, we incorporate the physical principles governing the optical system into the calibration process as a physics-based abstraction. This enables temperature prediction beyond the range covered by standard black body calibration, while accounting for the full spectral response of the imaging system. In particular, we refine the quantum efficiency (QE) values, typically reported in camera datasheets, using images acquired from a furnace that approximates a black body source at various temperatures. An overview of the full pipeline—from camera calibration and QE optimization to temperature prediction via a neural network—is illustrated in Figure 1.

The proposed method involves two main stages: a calibration phase for quantum efficiency (QE) optimization and a temperature estimation phase using a trained neural network. In the first stage, calibration images are acquired by imaging an Inconel 718 plate heated in a furnace across a known temperature range. An initial estimate of the camera’s spectral quantum efficiency is obtained from manufacturer datasheets. To account for the limited dynamic range of industrial cameras in the calibration phase, multiple images with varying exposure times at the same temperature are acquired. The calibration images are then preprocessed to extract measured radiation intensities, which are compared with simulated intensities based on Planck’s law and the initial QE estimate. A numerical optimization procedure is used to adjust the QE curve to minimize the discrepancy between measured and simulated data. The resulting optimized QE is subsequently used to simulate spectral radiation intensities over a wide temperature range (500−3500 °C), generating a synthetic dataset for the expected radiation intensities.

In the second stage, this synthetic dataset—consisting of simulated images paired with known temperatures—is used to train a neural network to perform temperature regression from normalized camera data. During inference, live images acquired by the camera are preprocessed similarly and input into the trained network, which outputs predicted thermal images. The neural network approach improves robustness to noise [21,34,45] and overcomes previous methods based on the approximation of the Planckian locus with polynomials [42,43,44]. Our method relies on normalization procedures that cancel all the effects that affect the magnitude of signal without affecting its spectral distribution, improving robustness towards exposure time or working distance variations while reducing the effect of the uncertainty on emissivity.

A wide experimental campaign validates the calibration method both on a standard color camera and on a multispectral camera, confirming that QE estimation is feasible on several cameras. Results of thermal measurements on calibration images are reported, with an estimate of their uncertainty. The generalization capability has been proven by measuring the temperature of a filament of a halogen lamp temperature calibrated. Our approach allows thermal imaging across a broad temperature range, including regions where the acquisition of calibration images at a known temperature is impractical, while ensuring consistency with the physical characteristics of the imaging system.

The paper is structured as follows. Section 2 describes the materials and methods used for the experiment including the cameras, the furnace for heat treatment of metals, and the quantum efficiency calibration method. Section 3 presents the experimental results obtained both with a color camera and with a multispectral camera. Section 4 discusses the results comparing them with the state of the art, while Section 5 presents some conclusions.

## 2. Materials and Methods

This section provides a detailed overview of the proposed method and experimental setup. Section 2.1 describes the industrial color camera used for the experiment, together with the furnace used for metal heat treatment, which approximates a black body source. The dataset acquisition procedure is detailed in Section 2.2. The full experiment has been repeated with a multispectral camera, introduced in Section 2.3.

Section 2.4 explains the physical modeling of the optical system, including the camera, lens, and filters. Initial data for the model hyperparameters are reported in Section 2.5. Section 2.6 describes the preprocessing of experimental data, and Section 2.7 introduces the calibration method used to refine the optical properties of the imaging system and to predict the Planckian locus in a wide temperature range. The neural network used for temperature inference—trained exclusively on a synthetic dataset describing the Planckian locus—is detailed in Section 2.8. Section 2.9 describes the method for uncertainty evaluation.

Finally, alternative temperature calibration methods from the literature are discussed in Section 2.10 and Section 2.11. The two-color method [17], which estimates temperature from the ratio of intensities at two wavelengths, is presented in Section 2.10. Another approach, presented in Section 2.11, derives the Planckian locus directly from quantum efficiency [36], offering insight into its physical significance. Calibration approaches specific to multispectral cameras are also discussed, particularly those involving the optimization of an anti-crosstalk matrix [34,35]. The results obtained with an anti-crosstalk matrix with the same camera used in this paper and the same furnace have been previously reported [34].

### 2.1. Camera and Lens

A general-purpose industrial color CMOS camera has been chosen for testing the calibration algorithm. The camera model is UI-3370CP-C-GL-R2 (IDS Imaging Development Systems GmbH, Obersulm, Germany). The maximum camera resolution is 2048 × 2048 pixels and pixel size is 5.5 μm. The optical chain includes a visible bandpass filter model BP550 (Midwest Optical Systems, Palatine, Illinois), used to reduce sensitivity outside the 400–700 nm range, and a 50 mm focal lens model LM50JC10M (Kowa Optimed Deutschland GmbH, Duesseldorf, Germany). The dark level offset is set to 50 to include 95% of the noise distribution of the signal generated by the dark current. The digital gain is set to the minimum and the sensor gain to −7 to maximize the dynamic range. Finally, a pseudo-white balance has been done by setting the gain of red, green, and blue channels to 1.0, 2.0, and 4.0, respectively.

### 2.2. Dataset Acquisition

**Figure 2:**
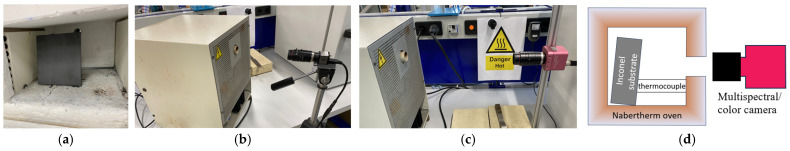
(**a**) Inconel 718 substrate inside the furnace. (**b**) Experimental setup for color camera calibration. (**c**) Experimental setup for multispectral camera calibration. (**d**) Sketch of the experimental setup.

The experimental setup (Figure 2) used for dataset acquisition includes a oven for heat treatments of metals capable of reaching 1100 °C (Nabertherm GmbH, Lilienthal, Germany). A hole in the lateral wall allows the camera to look inside the oven at an Inconel 718 substrate as shown in Figure 2. The inner chamber approximates a black body source of radiation.

The ground truth temperature of the substrate, a metallic plate of Inconel 718, is measured with a K thermocouple in contact with its surface. The thermocouple features mineral insulation and an Inconel 600 metallic sheath of 3 mm diameter. The maximum operating temperature is 1100 °C. Accuracy data are reported in Section 2.9. To ensure good thermal contact, the plate is slightly tilted to allow the thermocouple to rest firmly against its surface. The thermocouple is connected to a temperature controller that tunes the heating resistances of the oven.

The camera is positioned approximately 30 cm from the oven, with its optical axis roughly aligned with the axis of a viewing hole in the oven wall, providing a clear line of sight to the Inconel sample inside. The optics are focused on the surface of the Inconel plate, and the aperture is set to f/4.0.

Following the installation of the camera, the oven is switched on and set to its maximum operating temperature of 1100 °C. The heating process takes several hours, after which an additional hour is allowed to ensure thermal equilibrium between the oven, the sample, and the camera. Once thermal stability is achieved, a batch of images corresponding to 1100 °C is acquired.

Subsequently, the target temperature on the controller is decreased in 10 °C steps, down to 700 °C. The calibration temperature array is $T_{i}.$ At each step, a brief pause of a few minutes is observed to allow thermal stabilization before acquiring the next image batch. An additional acquisition is performed at 100 °C, where incandescence is negligible; these images serve as dark references, capturing sensor dark current across exposure times.

Ambient light is minimized during all measurements to avoid interference. For each temperature level, multiple exposure times are tested. For the color camera, the shortest exposure is 0.1 ms; for the multispectral camera, it is 10 ms. Each subsequent exposure is obtained by multiplying the previous one by approximately 1.26 (i.e., $10^{0.1}$), forming a logarithmic scale. This scale is chosen to match the exponential nature of blackbody radiation intensity as a function of temperature.

At each temperature and exposure setting, 10 images are acquired and averaged to reduce temporal noise. Dark-frame subtraction is applied using the 100 °C reference images. Demosaicing is then performed, and the average gray level for each spectral band is calculated within a predefined region of interest (ROI) on the Inconel 718 surface. The ROI corresponds to an elliptical area of approximately 10,000 pixels.

**Figure 3:**
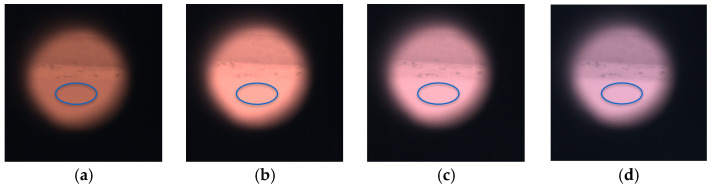
Calibration images of the inner part of the furnace acquired by the color camera: the Inconel 718 substrate is in the lower part. Exposure time and temperature from left to right are: (**a**) 0.5 ms at 1100 °C, (**b**) 5.0 ms at 950 °C, (**c**) 39.8 ms at 810 °C, and (**d**) 100.0 ms at 750 °C. The region of interest (ROI) is a blue ellipse.

The full dataset includes 13,330 images for the color camera and 5440 for the multispectral one. Figure 3 presents representative examples of the images acquired with the color camera: the circular field of view corresponds to the oven’s lateral aperture, with the surrounding area appearing dark. The Inconel 718 substrate is located at the lower portion of the frame, where the elliptical ROI is defined.

### 2.3. Multispectral Camera

**Figure 4:**
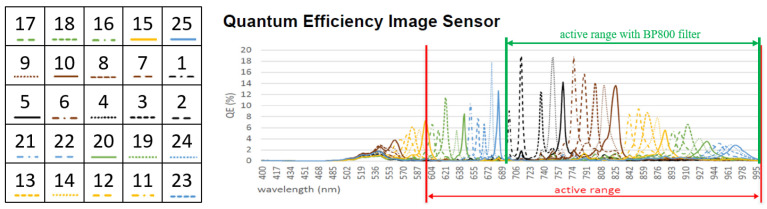
Demosaicing scheme and quantum efficiency (QE) from the datasheet of multispectral camera. The infrared pass BP800 filter limits the spectral active range to 700–1000 nm.

A second experimental campaign captured images of the same substrate with a multispectral camera in the near-infrared range. The used camera is MV1-D2048x1088-HS02-96-G2 (PhotonFocus AG, Lachen, Switzerland) based on the CMV2K-SM5x5-NIR sensor (Imec, Leuven, Belgium). The sensor has a pattern size of 5 × 5 with 25 different microfilters installed on the pixels. The quantum efficiency plot (Figure 4) shows a relevant spectral overlap of the QE among microfilters meaning that a photon of a specified wavelength could determine a count in multiple spectral bands. A BP800 bandpass filter (Midwest Optical Systems) has been installed to limit the sensitivity of the camera in the range 700–1000 nm and the same optic of the previous setup has been used. A neutral density filter ND200 (Midwest Optical Systems) has been installed to attenuate light by a factor of around 100 times. Images have been collected in the temperature range of 800–1100 °C with steps of 10 °C, since the attenuation filter made the images in the range 700–800 °C too dim. As in the previous case, 10 images are collected and averaged, dark images are subtracted, demosaicing is done according to the scheme in Figure 4, and the gray levels for each band and temperature are evaluated by averaging on an ROI corresponding to the metallic substrate.

### 2.4. Physical Modelling

**Figure 5:**
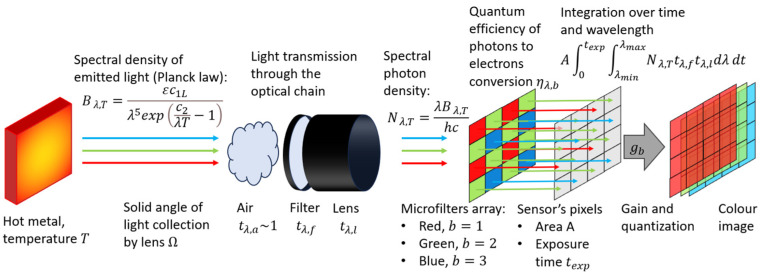
Physical modelling of light emission from incandescent metal, propagation through lens and filters, conversion into photoelectrons in the pixels of the sensors and postprocessing to obtain a color image.

Physical modelling of the imaging system is represented in Figure 5. The surface of the incandescent metal at temperature T emits radiation with spectral radiance described by the Planck law. Radiation attenuation by the transmission through air $t_{\lambda ,a}$ is considered negligible. Radiation is collected by the lens under a solid angle Ω and attenuated by the transmission through optical filters $t_{\lambda ,f}$ and through the lens $t_{\lambda ,l}$. The sensor converts photons into electrons, with quantum efficiency depending on band (red, green, blue) and wavelength $\eta_{\lambda ,b}$. Therefore, the Planck law is rewritten in terms of photon density. The pixel collects the photons hitting its sensitive area A and integrates them over exposure time $t_{exp}$ and over the entire spectral range. The photoelectron signal is then amplified by a band dependent gain $g_{b}$, quantized into an 8-bit digital signal and postprocessed to obtain a color image.

To estimate the number of photoelectrons generated in a camera pixel due to blackbody radiation, we begin with the spectral radiance of light $B_{\;\lambda ,T}$ emitted by a black body at temperature T and wavelength λ. The spectral radiance is described by Planck’s law and expressed in units of $Wm^{-2}sr^{-1}m^{-1}$. An emissivity coefficient ε, supposed to be independent of wavelength and temperature in graybody approximation, is introduced to describe the non-ideality of the emission. The spectral radiance is attenuated by considering the transmission spectra of the atmosphere $t_{\lambda ,a}$, of the lens $t_{\lambda ,l}$ and of the filters $t_{\lambda ,f}$.

(1) $$B_{\;\lambda ,T}=t_{\lambda ,a}t_{\lambda ,l}t_{\lambda ,f}\frac{\varepsilon c_{1L}}{\lambda^{5}exp\left(\frac{c_{2}}{\lambda T}-1\right)}.$$

The parameters $c_{1L}=2hc^{2}$ and $c_{2}=hc/k_{B}$ equal to 0.0144 mK [46], are the first and the second radiation constants, respectively. The spectral radiance is converted to spectral photon radiance $N_{\;\lambda ,T}$ by dividing by the energy of a single photon, E=hc/λ yielding the expression $N_{\;\lambda ,T}=\lambda B_{\;\lambda ,T}/(hc)$. The total number of photons incident on a pixel is then obtained by integrating this quantity over the sensor’s wavelength sensitivity range, and multiplying by the pixel area A, the solid angle of collection Ω, and the exposure time $t_{exp}$. To account for the detector’s efficiency, the integrand is weighted by the quantum efficiency $\eta_{\lambda ,b}$ of each band b (3 for the color camera and 25 for the multispectral one). The resulting number of photoelectrons $P_{b,T}$ collected by a pixel of band b at temperature T is thus given by Equation (2):

(2) $$P_{b,T}=A\Omega t_{exp}\int_{\lambda_{min}}^{\lambda_{max}}t_{\lambda ,a}t_{\lambda ,l}t_{\lambda ,f}\eta_{\lambda ,b}\frac{\lambda B_{\;\lambda ,T}}{hc}d\lambda .$$

The signal digitized by the camera $c_{b,T}$ is obtained by multiplying the photoelectrons $P_{b,T}$ by the internal gain of the camera $g_{b}$ that could be different for the red, green, and blue bands b due to the white balance. Dark signal subtraction allows to compensate for the contribution of dark current, and therefore its effect will be neglected in the simulation. The coefficient $K_{b}=Ag_{b}\Omega \varepsilon c_{1}/(hc)$ collects all the terms independent of wavelength and exposure time. Furthermore, the system quantum efficiency $q_{\lambda ,b}=t_{\lambda ,a}t_{\lambda ,l}t_{\lambda ,f}\eta_{\lambda ,b}$ is defined to keep into consideration all the terms depending on the wavelength. The digital signal collected by each pixel of the camera, after dark noise subtraction is reported in Equation (3):

(3) $$c_{b,T}=K_{b}t_{exp}\int_{\lambda_{min}}^{\lambda_{max}}\frac{q_{\lambda ,b}}{\lambda^{4}exp\left(\frac{c_{2}}{\lambda T}-1\right)}d\lambda .$$

To make color estimation versus temperature independent of exposure time, the whole Equation (3) is divided by $t_{exp}$, obtaining Equation (4):

(4) $$s_{b,T}=\frac{c_{b,T}}{t_{exp}}=K_{b}\int_{\lambda_{min}}^{\lambda_{max}}\frac{q_{\lambda ,b}}{\lambda^{4}exp\left(\frac{c_{2}}{\lambda T}-1\right)}d\lambda ,$$

where $s_{b,T}$ is the thermally generated signal simulated for a single pixel of the camera for band b and temperature T. The $K_{b}$ coefficients have to be optimized in the calibration step for each band independently. The $K_{b}$ coefficients are the product of internal gains $g_{b}$, related to camera configuration, emissivity ε dependent on the sample, AΩ depending on the experimental setup, and $c_{1}/(hc)$ that is constant. The gains $g_{b}$ are estimated in the calibration phase, while AΩ and ε are simplified by calculating the ratio of multiple bands.

### 2.5. Numerical Approximations

Integration is performed numerically by summing values calculated at selected sampling wavelengths. The integration range is chosen arbitrarily, considering where the optical system could show some sensitivity, with more attention to longer wavelengths where light emission by incandescent objects is stronger. The color camera shows sensitivity from UV to far infrared, and smooth QE versus wavelength. The multispectral camera has microfilters transmitting only narrow bands, and its sensitivity is in the near-infrared range, therefore a finer subsampling and a different integration range are considered. The integration parameters for the color and the multispectral cameras are reported in Table 1.

An initial estimate for the quantum efficiency (QE) $q_{\lambda ,\;b}$ of the system is obtained by multiplying the QE ${\tilde{q}}_{\lambda ,\;b}$ of each microfilter band for the transmission of the lens $t_{\lambda ,\;l}$ and of the filters $t_{\lambda ,\;f}$, as reported in Equation (5):

(5) $$q_{\lambda ,b}=\eta_{\lambda ,b}t_{\lambda ,l}t_{\lambda ,f}.$$

The transmission of the atmosphere $t_{\lambda ,a}$ is approximated to 1 since the system is designed mainly for applications in additive manufacturing, where the working distance is limited to a few tens of centimeters and the absorption of the air is negligible. Initial data are obtained from datasheets. For the multispectral camera, the QE reported in the datasheet (Figure 4) of each microfilter has been approximated by a Lorentzian–Cauchy distribution (intensity $\alpha_{b}$, half-width at half-maximum $\gamma_{b}$ and location parameter $\lambda_{b}$), multiplied by transmission of the filters $t_{\lambda ,f}$ and of the lens $t_{\lambda ,l}$ according to Equation (6):

(6) $$q_{\lambda ,b}=\frac{\alpha_{b}}{\pi \gamma_{b}\left(1+\left(\frac{\lambda -\lambda_{b}}{\gamma_{b}}\right)\right)}t_{\lambda ,l}t_{\lambda ,f}.$$

### 2.6. Data Preprocessing

**Figure 6:**
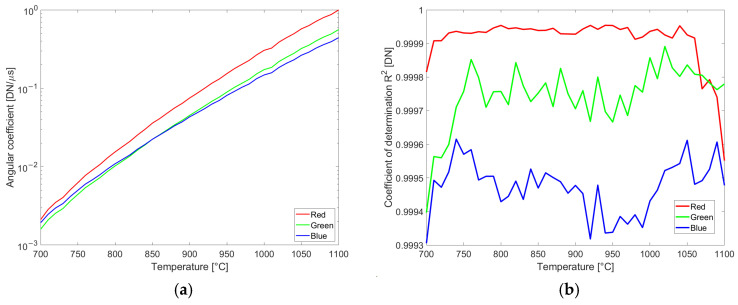
Results obtained by fitting gray levels of red, green, and blue bands of the color camera versus exposure time. (**a**) Angular coefficient of the fitting line of gray level (log scale) vs. temperature. (**b**) Coefficient of determination R^2^ versus temperature.

The goal of preprocessing is to prepare the experimental data $d_{b,T_{i},t_{exp}}$ representing the average gray values measured on calibration images versus band, temperature, and exposure time for the optimization step of $q_{\lambda ,b}$. Dividing the average gray value measured on the calibration images by exposure time is not accurate, due to the uncertainty in the dark level, even after dark image subtraction. Therefore, an alternative approach based on images acquired with multiple exposure times is proposed. First, the average gray values $d_{b,T_{i},t_{exp}}$ collected for the same temperature and the same band are grouped and plotted versus exposure time. Then, pixel intensities near saturation (here set above 240), and too dark (below 10), are discarded. On the remaining data, a least squares fitting of a line is performed. The angular coefficient $m_{b,T_{i}}$ is an accurate estimation of the thermally generated signal $s_{b,T_{i}}$ evaluated at the calibration temperatures $T=T_{i}$. Plots of angular coefficients and coefficients of determination R^2^ for the color camera are reported in Figure 6.

The proximity of the coefficient of determination R^2^ to 1 confirms the linearity of the sensor light collection and readout versus exposure time. The same range of values for R^2^ is obtained when processing data acquired with the multispectral camera.

### 2.7. Quantum Efficiency Optimization

The optimization algorithm improves the estimate of the QE by reducing the discrepancy between the simulated data $s_{b,T_{i}}$ (Section 3.1 and Section 3.2) and the experimental data $m_{b,T_{i}}$ (Section 3.3) for all calibration temperatures $T_{i}$ and bands. Optimization is designed to be performed independently on each band. Since coefficient $K_{b}$ is unknown, simulated data are divided by the datum at 1100 °C obtaining ${\overset{ˇ}{s}}_{b,T_{i}}=s_{b,T_{i}}/s_{b,1100{}^{\circ}C}$, and the same is done for measured data obtaining ${\overset{ˇ}{m}}_{b,T_{i}}=m_{b,T_{i}}/m_{b,1100{}^{\circ}C}$. The initial QE of the system composed by camera, lens, and infrared block filter is obtained from datasheets according to Equation (4) and identified as $q_{\lambda ,b}^{datasheet}$. QE is defined as an array of values, sampled on linearly spaced wavelengths with the step indicated in Table 1. The QE is scaled from the range 0–100% to 0–1. The iterative optimization process is an evolutive algorithm that modifies the QE array and keeps the new one if the loss function improves. Each optimization step starts by changing the value of the QE array by summing or subtracting a constant. If the result of the sum is outside the definition range of QE (0–1), the element of the array is set to the nearest limit (0 if negative and 1 if above). The modified QE array is used for $s_{b,T}$ estimation according to Equation (3), followed by division by the estimated intensity at 1100 °C to obtain ${\overset{ˇ}{s}}_{b,T_{i}}$. Then, the loss function is calculated. If the loss improves, the new QE array is kept, otherwise the modification is discarded. The optimization step is repeated on the next element of the array up to the end. To enforce convergence, the constant change is weighted on the wavelengths, with a weight equal to 0.01 in the transmission range of the optical filter (BP550 or BP800), decreasing gradually to 0.0001 outside. The optimization cycle is repeated for 400 iterations, decreasing the change by 10 times every 100 iterations on the entire array. The loss function is composed of the sum of two parts:

- The first part $L_{1}=rms_{b_{constant}}\left(\frac{{\overset{ˇ}{m}}_{b,T_{i}}}{{\overset{ˇ}{s}}_{b,T_{i}}}-1\right)$ checks that predicted intensities are correct for all the calibration temperatures $T_{i}$ for a specific band b;
- The second part verifies that the QE does not differ too much from the original one by the rms of the difference between estimated and nominal Qes.
- The optimization is repeated for the 3 bands of the color camera and for the 25 bands of the multispectral camera. The final QE of the system obtained at the end of the optimization is identified as $q_{\lambda ,b}^{optimum}$. Results are reported in Section 3.1. At the end of the optimization, for the color camera the simulated data has been divided by their maximum that is the value simulated for the red band at $T_{i}=1100{}^{\circ}C$ identified as $s_{1,1100{}^{\circ}C}$. The experimental data obtained by the color camera are also divided by their maximum $m_{1,1100{}^{\circ}C}$. After that the gain coefficients $K_{b}$ are estimated for each band as the ratio of the observed and simulated datum at 1100 °C and the simulated data are adjusted accordingly. The optimized quantum efficiency $q_{\lambda ,b}^{optimum}$ is used to simulate the Planckian locus relating color to temperature according to Equation (3).

### 2.8. Neural Network for Temperature Estimation

Temperature estimation is done through a regression neural network trained on a synthetic dataset corresponding to the Planckian locus previously calculated. The choice of training on full synthetic data is related to the fact that real training data with accurate ground-truth are difficult to obtain above 1100 °C—that is the maximum operating temperature of the black body source. Synthetic data enables robust, physics-informed training under controlled conditions. Results presented in Section 3.2 and Section 3.4 have been obtained by testing the network on real measurements showing the absence of bias.

Each image of the synthetic dataset has a width equal to 1 pixel and height equal to the number of bands of the camera, representing the simulated radiation intensities $S_{b,T}$ that could be observed at a specific temperature T. The training data, corresponding to the Planckian locus, are generated by Equation (3) with optimized QE $q_{\lambda ,b}^{optimum}$ providing $s_{b,T}$. Simulated data $s_{b,T}$ are normalized to cancel the dependence on emissivity, exposure time and experimental setup. The training data for the color camera are stored in an array of two-element tuples obtained by dividing the red and blue simulated data by green $S_{b,T}=\left(\frac{s_{1,T}}{s_{2,T}},\frac{s_{3,T}}{s_{2,T}}\right)\;$. The green over green ratio, equal to 1, is discarded. Equation (7) shows that normalization of simulated data cancels the effects of the optical configuration AΩ and of the emissivity ε in a graybody approximation.

(7) $$S_{1,T}=\frac{s_{1,T}}{s_{2,T}}=\frac{\frac{A\Omega g_{1}\varepsilon c_{1}}{hc}\int_{\lambda_{min}}^{\lambda_{max}}\frac{q_{\lambda ,b}}{\lambda^{4}exp\left(\frac{c_{2}}{\lambda T}-1\right)}d\lambda }{\frac{A\Omega g_{2}\varepsilon c_{1}}{hc}\int_{\lambda_{min}}^{\lambda_{max}}\frac{q_{\lambda ,b}}{\lambda^{4}exp\left(\frac{c_{2}}{\lambda T}-1\right)}d\lambda }=\frac{g_{1}\int_{\lambda_{min}}^{\lambda_{max}}\frac{q_{\lambda ,b}}{\lambda^{4}exp\left(\frac{c_{2}}{\lambda T}-1\right)}d\lambda }{g_{2}\int_{\lambda_{min}}^{\lambda_{max}}\frac{q_{\lambda ,b}}{\lambda^{4}exp\left(\frac{c_{2}}{\lambda T}-1\right)}d\lambda }.$$

Furthermore, $S_{1,T}=\frac{s_{1,T}}{s_{2,T}}=\frac{c_{1,T}}{t_{exp}}\frac{t_{exp}}{c_{2,T}}=\frac{c_{1,T}}{c_{2,T}}$; therefore, the effect of exposure time is also canceled. For the multispectral camera simulated data $s_{b,T}$ are normalized by dividing by the average of the bands in the mid-spectral range (bands from 9 to 16). The dataset consists of 3000 synthetic tuples, generated at 1 °C intervals over a temperature range from 500 °C to 3500 °C. The dataset has been randomly split into 70% for training and 30% for testing. Synthetic data are used because accurate ground-truth measurements are difficult to obtain across the full temperature range up to 3500 °C. The use of synthetic data allows consistent, noise-free labels for training, making them ideal for developing and validating the regression network. The network is a multilayer perceptron with 5 layers (250, 200, 150, 100, and 50 neurons per layer with ReLU activation).

Loss is cross entropy L2 and no dropout is used. L2 loss is chosen because it is well suited for regression tasks such as temperature estimation. It penalizes larger errors more heavily than smaller ones, thereby encouraging the model to produce accurate predictions across the entire temperature range. Moreover, it provides smooth gradients and promotes stable convergence during optimization, which is important for training convergence. Since the training is conducted exclusively on synthetic data, free from noise or experimental variability, there is no need to employ noise-robust alternatives such as L1 or Huber loss. The training is executed on an Intel CPU for 400 epochs using a Adam optimizer with a learning rate of 0.00001 and a batch size of 32.

In inference, the network accepts as input gray value data, normalized as the simulated ones, and predicts a temperature. Normalization of gray value data removes, at the first order of approximation, all the contribution that do not alter the wavelength spectrum of the signal including exposure time, optics aperture, working distance, and reduce the effect of uncertainty on emissivity.

The results obtained by the proposed approach are compared, in Section 3, with the results obtained with two state-of-the-art methods, described in Section 2.9 and Section 2.10.

### 2.9. Uncertainty Evaluation

To ensure the traceability and reliability of the temperature estimation, an uncertainty budget is evaluated according to the Guide to the Expression of Uncertainty in Measurement (GUM) principles. The uncertainty on the estimated temperature is evaluated by propagating the uncertainties of the input parameters through the calibration model using the law of propagation of uncertainty. According to the approximation of independence between noise sources, the sensitivity coefficients of the temperature estimation function with respect to each uncertain parameters—such as camera noise and emissivity slope—are determined. These are combined with the standard uncertainties of each parameter to compute the combined standard uncertainty.

The temperature estimation process includes the following steps: image acquisition, dark image subtraction, normalization, and neural network regression. After dark subtraction, the resulting signal in each band is denoted $d_{b}$. The final estimated temperature $T_{est}$ is expressed as a differentiable function $f_{cal}$ (representing normalization and neural network regression) that is optimized during calibration. Therefore $T_{est}=f_{cal}\left(d_{1},d_{2},\ldots \right)$. In the hypothesis that the signals over different bands presents non-correlated noise sources; the total uncertainty is given by the partial derivative of the calibration model $f_{cal}$ versus the signal $d_{b}$ for each band multiplied by the uncertainty on the same band $u{\left(d_{b}\right)}^{2}$ plus the uncertainty affecting the calibration model.

The main uncertainty sources affecting the calibration model itself are uncertainty on emissivity slope $\varepsilon_{1}$ and on reference temperature. Zero-order emissivity effects are removed through normalization, but the residual dependence on emissivity slope remains. The uncertainty on emissivity slope has been estimated considering how emissivity could change among several materials. The contribution of image noise to the calibration process is considered negligible due to the averaging of 10 images over a region of 10,000 pixels, reducing the variance by a factor of $10^{5}$. The uncertainty of the reference temperature $u\left(T_{ref}\right)$ is determined by the thermocouple’s intrinsic uncertainty (±4.4 °C, IEC 60584-2 Class 1), supposing a rectangular distribution due to the limited available data. The sensitivity coefficient $\alpha_{T_{ref}}$ describing the propagation of uncertainty on reference temperature through the calibration model has been estimated by the Montecarlo method. The partial derivatives method appeared not applicable since uncertainty on reference temperature affects several parameters that are correlated by the calibration model. Contributions to the uncertainty are summarized in Table 2.

The temperature measurement relies on an image acquired by a color camera (sensor CMV4000 by CMOSIS) and by a multispectral camera (CMV2000 by CMOSIS), making the image acquisition process a central contributor to uncertainty. The sensor of the color camera has a saturation capacity of $\mu_{e,sat}=8389\;e^{-}$, and a readout noise variance of $\sigma_{d}^{2}=15e^{-}$. The sensor of the multispectral camera has a saturation capacity of $\mu_{e,sat}=13.5\;ke^{-}$, and a readout noise variance of $\sigma_{d}^{2}=13e^{-}$. According to the EMVA 1288 standard, the digital output signal $\mu_{y}$ is given by: $\mu_{y}=G_{b}(\mu_{e}+\mu_{d})$ where $\mu_{e}$ is the number of photoelectrons, $\mu_{d}$ is the dark current, and $G_{b}$ is the internal gains for band b. The total variance of the digital signal is $\sigma_{y}^{2}=G_{b}^{2}\left(\sigma_{d}^{2}+\sigma_{e}^{2}\right)+\sigma_{q}^{2}$ where $\sigma_{e}^{2}=\mu_{e}$ is the contribution of shot noise with Poisson distribution; $\sigma_{d}^{2}$ is noise related to sensor read out signal, and $\sigma_{q}^{2}$ is quantization error. For high signal levels and short exposure times, shot noise dominates, leading to a signal to noise ratio (SNR) on the color camera of 92 corresponding to a standard deviation of noise equal to 2.78 DN. The SNR of the multispectral camera is 116, corresponding to a standard deviation of noise equal to 2.19 DN. The noise quantization, considering that the pixel bit depth is 8 bit for both cameras, has a standard deviation of $\frac{255\;DN}{2^{8}\sqrt{12}}$ approximately equal to 0.29 DN, and is independent on exposure time and gain.

The full uncertainty budget is presented in Equation (8).

(8) $$u\left(T_{est}\right)=\sqrt{\sum_{b}{\left(\frac{\partial f_{cal}}{\partial d_{b}}u\left(d_{b}\right)\right)}^{2}+{\left(\alpha_{T_{ref}}u\left(T_{ref}\right)\right)}^{2}+{\left(\frac{\partial f_{cal}}{\partial \varepsilon_{1}}u\left(\varepsilon_{1}\right)\right)}^{2}+u{\left(f_{cal}\right)}^{2}}.$$

The partial derivatives $\frac{\partial f_{cal}}{\partial d_{b}}$ have been estimated numerically by running the temperature prediction model on synthetic data according to Equation (9):

(9) $$\frac{\partial f_{cal}}{\partial d_{b}}\approx \frac{f_{cal}\left(d_{b}\right)-f_{cal}\left(d_{b}+\Delta \right)}{\Delta }.$$

For the partial derivatives involving camera signal, the parameter Δ has been set to 1 DN. For the partial derivatives involving emissivity slope, the parameter Δ has been set to 0.01 $\mu m^{-1}$. The sensitivity coefficient on temperature has been evaluated by running the full calibration model after applying a noise distribution with the described characteristics to the reference temperatures. To determine the propagation on reference temperature through the calibration model, the Montecarlo method has been used. Several optimization runs of the QE have been done to estimate the propagation of uncertainty on the reference temperature through the calibration model.

Results of uncertainty versus temperature for the color and the multispectral camera are reported in Section 3.5.

### 2.10. Two-Color Method

The most widely experimented approach in the state of the art for estimation of temperature from color cameras consists of approximating the red and the green channels as narrow bands and applying the ratio pyrometer formula [13]. This method completely neglects both the spectral overlaps of the red and green channels and the infrared light in the range from 700 to 1000 nm collected by the sensor due to imperfections in the infrared cut filter. One of the latest implementations of the two-color approach partially solves these issues [17]: in the hypothesis that temperature is below 2000 K the intensity of the blue channel is supposed to be mainly due to infrared light above 700 nm. Therefore, dimensionality reduction is applied as $m_{1,T_{i}}-m_{3,T_{i}}$ and $m_{2,T_{i}}-m_{3,T_{i}}$. The blue channel $m_{3,T_{i}}$ is subtracted to the red $m_{1,T_{i}}$ and to the green ones $m_{2,T_{i}}$ to reduce the infrared light component. To keep spectral overlap in consideration, the wavelengths of the red and green channels, $\lambda_{red}^{eq}$ and $\lambda_{green}^{eq}$, are chosen by an optimization procedure. The formula for temperature estimation (in °C) is:

(10) $$T_{2color}\left(T_{i}\right)=\frac{c_{2}\left(\frac{1}{\lambda_{green}^{eq}}-\frac{1}{\lambda_{red}^{eq}}\right)}{5ln\left(\frac{\lambda_{red}^{eq}}{\lambda_{green}^{eq}}\right)+ln\left(r_{4}\frac{r_{1}m_{1,T_{i}}-r_{3}m_{3,T_{i}}}{{r_{2}m}_{2,T_{i}}-r_{3}m_{3,T_{i}}}\right)}-273.15\;K.$$

In Equation (10) the estimated temperature is $T_{2color}$ while $c_{2}$ is the second radiation constant. Calibration requires two steps. The first step has the goal to estimate the gains $r_{b}$ applied to the measured values. Gains are determined by plotting the measured data $m_{b,T_{i}}$ versus the data $s_{b,T_{i}}$ simulated with datasheet QE obtaining three plots, one for each band b. In each plot the data are fitted with a line: the intercept is negligible and the gain for each band $r_{b}$ is calculated as the inverse of the angular coefficient. The procedure is then repeated by comparing normalized data after blue subtraction with simulated ones determining the gains ratio $r_{4}$. Once the gains are optimized the second calibration step optimizes equivalent wavelengths $\lambda_{red}^{eq}$ and $\lambda_{green}^{eq}$ by a non-linear minimization. The goal function is the root mean square of the temperature prediction error $rms(T_{2color}\left(T_{i}\right)-T_{i})$ for all the calibration temperatures $T_{i}$. Initial optimization values are 500 nm for green and 620 nm for red. Results are reported in Section 3.6.

### 2.11. Effect of Quantum Efficiency

To investigate the impact of quantum efficiency on temperature estimation, an additional literature method for calibrating the temperature of a color camera is considered. This approach aims to estimate the Planckian locus directly from QE [36], mapping color to temperature in a two-dimensional color space. Temperature is then regressed from color using Ohno’s interpolation method [44]. Estimated color data $s_{b,T}$ are obtained according to Equations (3) and (4) with the quantum efficiency dataset $q_{\lambda ,b}$ obtaining $s_{b,T_{i}}(q_{\lambda ,b})$. The measured data $m_{b,T_{i}}$ for each band are adjusted to match the simulated values by multiplying them by the $u_{b}$ coefficients. These gains are calculated as the ratio $u_{b}=s_{b,1100{}^{\circ}C}/m_{b,1100{}^{\circ}C}$, where $s_{b,1100{}^{\circ}C}$ represents the simulated value at 1100 °C and $m_{b,1100{}^{\circ}C}$ is the corresponding measured value. After determining gains $u_{b}$ both the simulated and the measured data are converted into chromaticity coordinates, obtaining a two-dimensional representation, which describes color independent of luminance. Experimental data coordinates are $M_{1,T_{i}}=\frac{u_{1}m_{1,T_{i}}}{\sum_{b}u_{b}m_{b,T_{i}}}$ and $M_{2,T_{i}}=\frac{u_{2}m_{2,T_{i}}}{\sum_{b}u_{b}m_{b,T_{i}}}$ while simulated data coordinates are $S_{1,T_{i}}=\frac{s_{1,T_{i}}}{\sum_{b}s_{b,T_{i}}}$ and $S_{2,T_{i}}=\frac{s_{2,T_{i}}}{\sum_{b}s_{b,T_{i}}}$. The temperature $T_{QE}\left(T_{i}\right)$ is estimated for each value of $T_{i}$ by mapping the experimental coordinates $\left(M_{1,T_{i}},M_{2,T_{i}}\right)$ onto the Planckian locus defined by $\left(S_{1,T_{i}},S_{2,T_{i}}\right)$ with triangular interpolation [44]. The simulation has been repeated with the QE obtained by the datasheet $q_{\lambda ,b}^{datasheet}$ and with the QE optimized from our method $q_{\lambda ,b}^{optimum}$. To take into account some infrared absorption not considered by $q_{\lambda ,b}^{datasheet}$, the simulation has been repeated by increasing $q_{\lambda ,b}^{datasheet}$, mapped between 0 and 1, by 0.01, 0.02, and 0.05. Results are reported in Section 3.6.

## 3. Results

Results regarding QE optimization (Section 3.1), temperature estimation on calibration images (Section 3.2), and the capability of generalizing outside the calibration range are introduced in Section 3.3. Results obtained with alternative calibration methods are reported in Section 3.4.

### 3.1. Results for Optimized Quantum Efficiency

**Figure 7:**
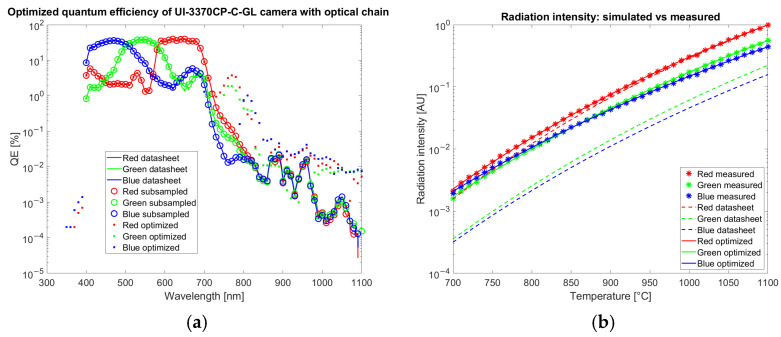
Results of the optimization of quantum efficiency for the color camera. (**a**) QE from the datasheet, subsampled, and optimized for red, green, and blue bands versus wavelength. (**b**) Measured and simulated radiation intensities for red, green, and blue versus temperature. The results of simulations based on both the datasheet QE and the optimized one are reported.

**Figure 8:**
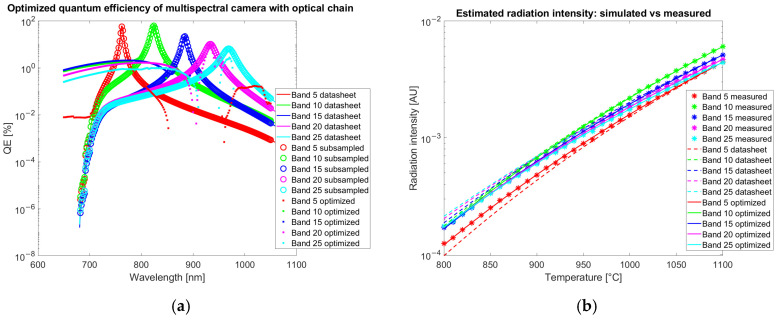
Results of the optimization of QE for the multispectral camera for bands 5,10,15,20, and 25. (**a**) The QE versus wavelength: from the datasheet, subsampled, and optimized. (**b**) Measured and estimated radiation intensities versus temperature. Estimates based on both datasheet and optimized QEs are reported.

The results of the optimization of QE for the color camera are reported in Figure 7 and for the multispectral camera in Figure 8. The radiation intensities are estimated with Equation (3) and corrected by the gain coefficient $K_{b}$. Estimation is repeated both with QE from the datasheet $q_{\lambda ,b}^{datasheet}$, and with optimized QE $q_{\lambda ,b}^{optimum}$.

Data reported in Figure 7a shows that the correction applied to the QE by the optimization is around 1% from 700 to 900 and about 0.01% from 900 to 1100 nm where 100% corresponds to the collection of all the incoming photons. On average, the quantum efficiency increased by 0.05%. The rms of the difference in the QE before and after the optimization is 0.66%. After optimization the RMS of the percentage error between measured and theoretical values is 2.3% on the entire dataset.

The correction applied to the QE of the multispectral camera (Figure 8a) by the optimization algorithm is around 1% or lower.

Both for the color and the multispectral camera, the optimization strongly improves the agreement of simulated data with experimental measurements acquired in calibration as observed in Figure 7b and Figure 8b (crosses corresponding to measured data are superimposed to the continuous line calculated with an optimized QE and not to dashed lines obtained from datasheet QE).

**Figure 9:**
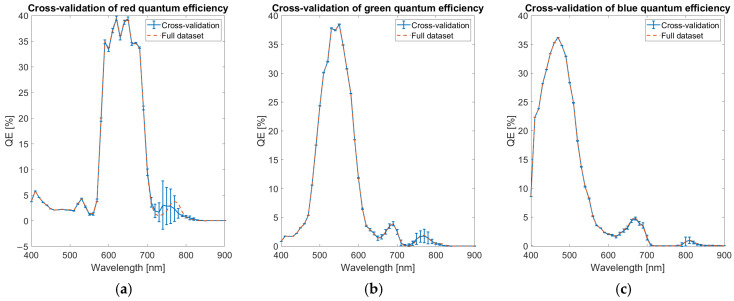
(**a**) Cross validation of quantum efficiency estimation for the red band of color camera. (**b**) Cross validation of quantum efficiency estimation for the green band of color camera. (**c**) Cross validation of quantum efficiency estimation for the blue band of color camera.

To assess the robustness of the QE estimation, a cross-validation procedure is performed. The calibration dataset is divided into five distinct temperature groups, uniformly sampled across the available temperature range. The QE optimization model is independently trained on each of these subsets, resulting in five separate QE curves. The average and standard deviation across these curves are then computed at each wavelength, providing an estimate of the uncertainty associated with the QE determination. This wavelength-dependent uncertainty is reported as error bars with coverage factor of 1 in the cross-validation QE plots in Figure 9, offering an insight into the sensitivity of the calibration process to variations in the calibration data. The cross-correlation confirms the robustness of the QE estimation algorithm with only limited variations of a few percent in the spectral region among 700 and 850 nm, where the leaks of the infrared-cut filters are present.

### 3.2. Temperature Estimation in the Calibration Range

The results of the training of the neural network for temperature estimation are reported in Table 3. Images are resized by a factor of two to avoid interpolation due to demosaicing. The network is tested and trained on the synthetic dataset simulated with optimized quantum efficiency. The test has been repeated by estimating temperature from single pixels of calibration images.

**Figure 10:**
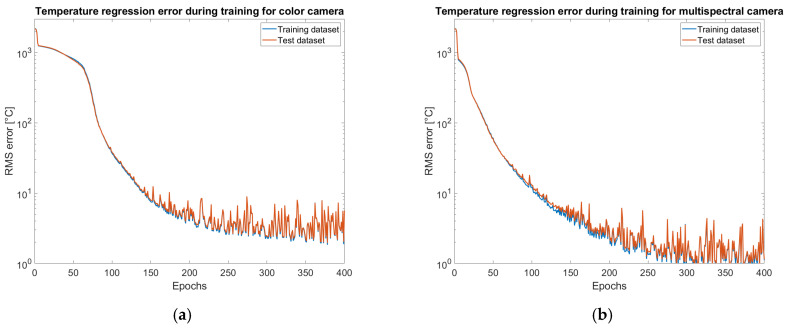
(**a**) Training and validation loss curves over epochs for the color camera. (**b**) Training and validation loss curves over epochs for the multispectral camera.

The regression errors evaluated on training and test datasets have been recorded during the training at the end of each epoch. The plots or rms error on temperature prediction versus the training epoch reported in Figure 10 show a smooth convergence behavior, with no signs of overfitting. The model achieves consistent generalization performance across the test dataset.

**Figure 11:**
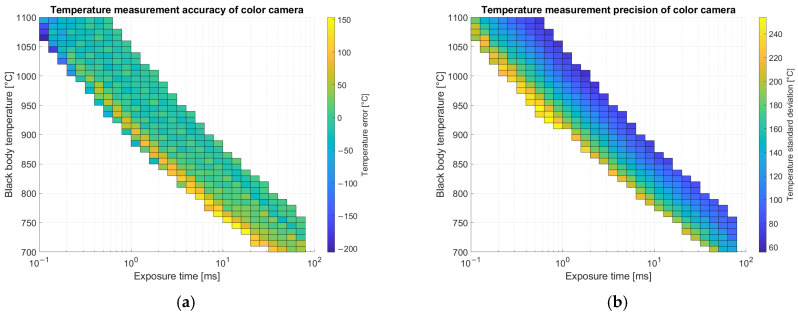
(**a**) Temperature measurement error on calibration images for the color camera. The white regions do not report temperature measurements since the corresponding images are too dim or saturated. (**b**) The standard deviation of temperature measurements inside the region of interest on calibration images for the color camera.

**Figure 12:**
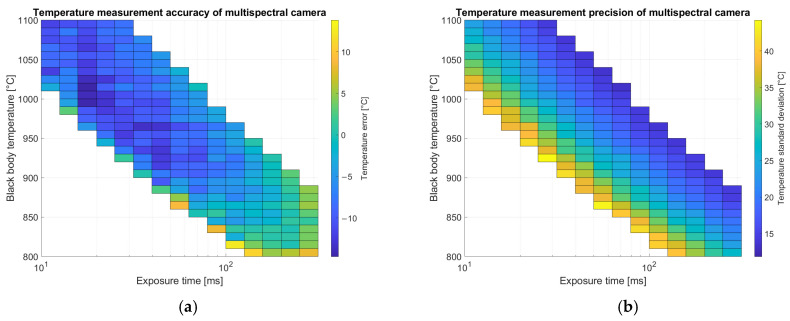
(**a**) Temperature measurement error on calibration images for the multispectral camera. White regions do not report temperature measurements, since the corresponding images are too dim or saturated. (**b**) The standard deviation of temperature measurement inside the region of interest on calibration images for the color camera.

Inference is done by generating a temperature image pixel by pixel. The first step is to subtract the dark image, then demosaicing is applied and images of the single channels are split. For pixels non-saturated on all the bands (20–220 digital numbers), an estimator independent of exposure time is evaluated. For the color camera both red over green and blue over green ratios are chosen. The normalized estimators are provided to the network that regresses a temperature measurement. Results for average error and standard deviation on calibration images for the color camera are shown in Figure 11 and for the multispectral camera in Figure 12.

Results reported in Figure 11 for temperature measurements are related only to images with a relevant number of valid pixels (not saturated nor dim) in the Region of Interest (ROI) corresponding to the metallic substrate shown in Figure 3 as a blue ellipse. In the ROI, supposed at uniform temperature, average and standard deviation of predicted temperatures over the pixels are evaluated. Accuracy is estimated as the difference between the measured temperature on each pixel and the ground truth measured with the thermocouple, averaged on the entire ROI. Pixel-to-pixel variations evaluated by standard deviation provide an estimate of precision. Accuracy is better on images with exposure times that are intermediate between the minimum and the maximum allowed for that temperature. On the other hand, precision (standard deviation) is lower when the exposure time is higher due to the better signal-to-noise ratio.

Results reported in Figure 12 for temperature measurements with the multispectral camera are related only to images with a relevant number of valid pixels (not saturated nor dim) in the Region of Interest (ROI) corresponding to the metallic substrate. Accuracy shows less dependence on exposure time with respect to the color camera but depends slightly on temperature. Precision is better on images with higher exposure time due to the better signal-to-noise ratio.

### 3.3. Results About Electronic Noise

The plots reported in Figure 11b and Figure 12b reports the standard deviation of the estimated temperature versus reference temperature and exposure time for color camera and multispectral camera, respectively. They both follow a similar pattern showing that, at a fixed reference temperature, the standard deviation of the predicted temperature in a unform region reduces together with exposure time. This behavior seems to highlight a strict relation between exposure time, reference temperature, and uncertainty related mainly to the electronic noise of the camera. To verify this assumption a modelling of electronic noise versus exposure time is proposed. According to EMVA 1288 the variance of the noise $\sigma_{y}^{2}$ could be obtained by summing shot noise, readout noise and quantization error $\sigma_{y}^{2}=G_{b}^{2}\left(\sigma_{d}^{2}+\sigma_{e}^{2}\right)+\sigma_{q}^{2}$ as explained in Section 2.9. The signal generated by the camera is $d_{b}=G_{b}\mu_{eb}$, considering the dark subtraction. The normalization calculates the ratio of couples of signals: red ($d_{1})$ over green ($d_{2})$ corresponding to $d_{1}/d_{2}$ and blue ($d_{3})$ over green corresponding to $d_{3}/d_{2}$. Therefore, according to uncertainty propagation, the total noise variance on the red to green ratio could be calculated according to Equation (11):

(11) $$\sigma_{d1/d2}^{2}=\frac{\sigma_{\mu_{y1}}^{2}}{d_{2}^{2}}+\frac{\sigma_{\mu_{y2}}^{2}}{d_{2}^{4}}\mu_{y1}^{2}.$$

By considering that the photoelectrons depends on photon flux $\phi_{pb}$, by quantum efficiency $\eta_{b}$ and by exposure time $t_{exp}$, the signal could be expressed as $d_{b}=G_{b}t_{exp}\eta_{b}\phi_{pb}$. Since the shot noise has a Poisson distribution its variance could be expressed in terms of photoelectron signal $\sigma_{e,b}^{2}=\mu_{e,b}=$ $t_{exp}\eta_{b}\phi_{pb}$. Substituting in Equation (11) and grouping versus exposure time Equation (12) is obtained:

(12) $$\sigma_{1/2}^{2}=\frac{1}{t_{exp}^{2}}\left[\frac{K_{1}^{2}\sigma_{d}^{2}+\sigma_{q}^{2}}{{\left(K_{2}\phi_{p2}\eta_{2}\right)}^{2}}+\frac{K_{2}^{2}\sigma_{d}^{2}+\sigma_{q}^{2}}{{\left(K_{2}\phi_{p2}\eta_{2}\right)}^{4}}{\left(K_{1}\phi_{p1}\eta_{1}\right)}^{2}\right]+\frac{1}{t_{exp}}\left[\frac{K_{1}^{2}\phi_{p1}}{{\left(K_{2}\phi_{p2}\right)}^{2}}+\frac{\eta_{1}^{2}\phi_{p2}}{{{\eta^{2}K}_{2}^{2}\left(\phi_{p2}\right)}^{4}}{\left(K_{1}\phi_{p1}\right)}^{2}\right].$$

Equation (12) could be simplified as $\sigma_{1/2}^{2}=t_{exp}^{-2}A_{1/2}+t_{exp}^{-1}B_{1/2}$ by defining a first term $A_{1/2}$ that multiplies $t_{exp}^{-2}$ dependent on electronic noise, and a second term $B_{1/2}$ that multiplies $t_{exp}^{-1}$ independent of electronic noise but dependent only on ratios of gains, quantum efficiencies, and photon fluxes. The total noise variance is the sum of the variances over the two ratios weighted by sensitivity coefficients determined by propagation through the neural network. Total variance is therefore $\sigma_{tot}^{2}=\sigma_{1/2}^{2}\alpha_{1/2}^{2}+\sigma_{3/2}^{2}\alpha_{3/2}^{2}$. Substituting the results obtained for $\sigma_{1/2}^{2}$ and $\sigma_{3/2}^{2}$ and multiplying by $t_{exp}^{2}$ the Equation (13) is obtained:

(13) $$\sigma_{tot}^{2}t_{exp}^{2}=\left(B_{1/2}\alpha_{1/2}^{2}+{B_{3/2}\alpha }_{3/2}^{2}\right)t_{exp}+\left(A_{2/3}\alpha_{2/3}^{2}+{A_{2/3}\alpha }_{2/3}^{2}\right).$$

**Figure 13:**
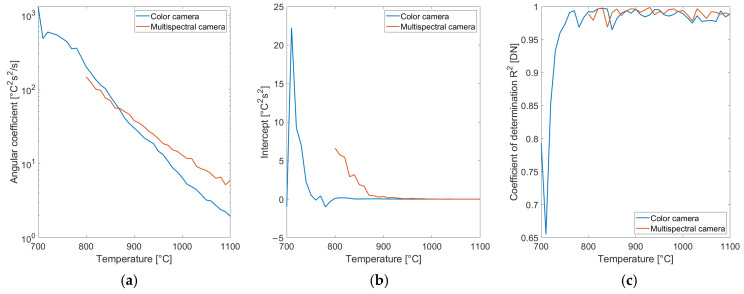
(**a**) Semilog plot of angular coefficient, (**b**) line intercept, and (**c**) coefficient of determination obtained by line fitting the measured temperature standard deviation versus exposure time according to the model presented in Equation (13).

Equation (13) corresponds to a line. In order to verify the correctness of the modeling the standard deviation data reported in Figure 11b and Figure 12b are grouped by reference temperature. The data in each group are squared, multiplied by the square of the exposure time and regressed to a line versus exposure time. Fitting results for the color and the multispectral cameras are reported in Figure 13 versus reference temperature.

The results reported in Figure 13 confirm the validity of the uncertainty model proposed to account for the electronic noise since the coefficient of determination is nearly one for almost all temperatures except the lowest ones. The model points out the relevant role of the exposure time in the uncertainty. The line intercept, keeping into account the effect of electronic noise is almost negligible for temperatures above 800 °C for the color camera and at 900 °C for the multispectral one, showing the limited contribution of this term. The largest contribution to the noise is provided by the angular coefficient, taking into account the imbalance in the photon flux over several channels.

### 3.4. Temperature Estimation Outside the Calibration Range

The temperature estimation network is trained using simulated data covering a temperature range of 500–3500 °C for both a color camera and a multispectral camera. To evaluate its ability to generalize beyond the calibration range (700–1100 °C), images of a temperature-calibrated halogen bulb (Stefan Boltzmann lamp by 3B Scientific) are captured and processed using both cameras.

The filament temperature is adjusted by controlling the applied voltage, ranging from 1.5 V (corresponding to 800 °C, where incandescence becomes visible) to a maximum of 12.0 V (2600 °C), which represents the filament’s nominal operating condition. Temperature is calculated from the electrical resistance of the filament. Images are captured at temperature increments of 100 °C, from 800 °C up to 2600 °C, setting the corresponding voltage on the tunable power supply.

For each temperature level, multiple exposure times are used, matching those applied during image acquisition at the furnace, extended to reach the minimum exposure time allowed by both cameras (40 μs for the color camera and 50 μs for the multispectral). At each exposure time and voltage, five images are captured and averaged to reduce quantization error. The dark signal is corrected by subtracting the average of five images taken with the same exposure time but with the lamp turned off.

A rectangular region of interest (ROI) is defined in the image over the filament. Pixels with gray values exceeding 240 (before dark subtraction) are considered saturated and removed, as well as those with gray values below 10. If more than half of the pixels in the ROI remain after filtering, the image is processed by evaluating the temperature for each individual pixel.

**Figure 14:**
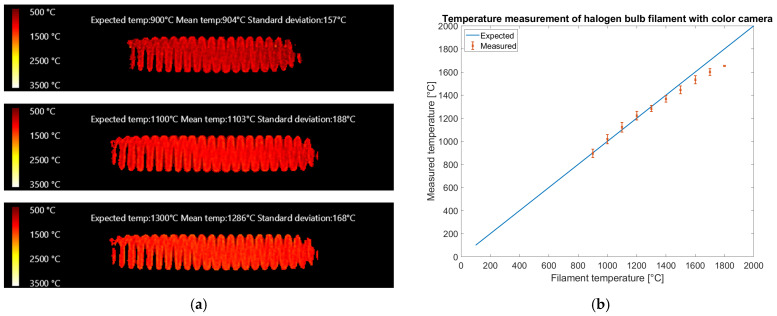
(**a**) Temperature images of the filament at 900 °C, 1100 °C, and 1300 °C. Exposure times are 15.8 ms, 3.16 ms, and 0.5 ms. (**b**) Average temperature measurements of the halogen bulb filament from all the images captured by the color camera at the same supply voltage, compared to the reference temperature from the lamp datasheet. The error bars represent twice the standard deviation over mean temperatures measured on images with the same filament temperature but different exposure times.

Temperature images of the filament are shown in Figure 14a, reporting expected temperature, mean temperature, and standard deviation evaluated on all the valid pixels of the region of interest. Figure 14b presents a plot of the average temperature estimates for images taken at the same halogen bulb voltage but with different exposure times. The error bars represent twice the standard deviation over mean temperatures measured on images with the same filament temperature but different exposure times.

The results reported in Figure 14 show that the color camera could measure up to 1800 °C: above that value, the red band saturates at the shortest exposure time allowed by the camera (40 μs) and color-to-temperature conversion is not possible. The reference temperature is within error bars up to 1400 °C, showing that our calibration method generalizes above the calibration range (700–1100 °C). Between 1400 °C and 1800 °C, the temperatures appear underestimated. The rms error on filament temperature measurements on the reduced dataset in the expected temperature range of 900–1400 °C is 28 °C on 40 valid filament images, showing enough pixels were neither saturated nor dim. The rms error on the full temperature range including 59 valid images is 58 °C.

**Figure 15:**
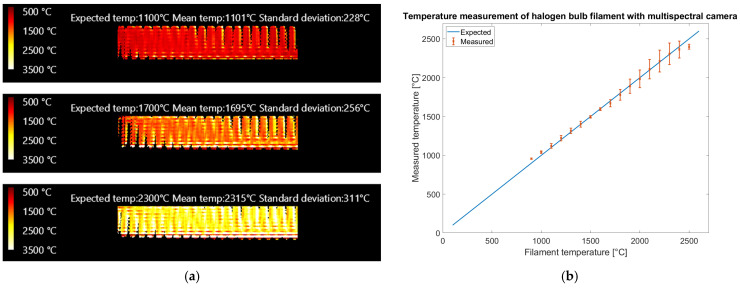
(**a**) Temperature images of the filament at 1100 °C, 1700 °C, and 2300 °C. Exposure times are 10.0 ms, 0.5 ms, and 158 μs. (**b**) Average temperature measurements of the halogen bulb filament from all the images captured by the multispectral camera at the same supply voltage, compared to the reference temperature from the lamp datasheet. Error bars indicate standard deviation among several temperature images due to non-optimal exposure time.

Figure 15 presents the results obtained with the multispectral camera.

The results reported in Figure 15 show that the multispectral camera could measure up to 2600 °C, although uncertainty starts increasing above 1800 °C due to the saturation of some bands. Different from the color camera, a limited number of saturated bands have been accepted in the processing at the price of higher uncertainty. The reference temperature is within error bars up to 2400 °C, showing that our calibration method generalizes above the calibration range (700–1100 °C). The rms error on filament temperature prediction, evaluated on the entire dataset of 123 valid images up to 2600 °C, showing at least 50% of the filament neither saturated nor dimmed, is 45.8 °C. Limiting the temperature range to 1000–1400 °C, as the color camera, 46 valid images remain with rms error on temperature prediction reduced to 31.0 °C.

The color camera generates temperature images (Figure 14a) with higher spatial resolution than the multispectral camera (Figure 15a), allowing the visualization of the wires of the coil. This could be explained considering that the pixel size of both cameras is 5.5 μm and that the magnification provided by the lens is roughly the same, but the multispectral camera requires a subregion of 5 × 5 pixels of the sensor to generate a temperature read while the color camera, thanks to the debayering algorithm with interpolation, generates a temperature read for every pixel. On the multispectral camera, the distance among sampling regions for the different bands is much bigger than on the color camera, and this could be critical when sharp temperature edges are present. In the multispectral camera the bands in the subregion used for temperature estimation may observe regions of the sample at different temperatures, potentially generating false temperature readings. Nonetheless the operating range of the multispectral camera is wider, covering the temperature range from 1000 °C to 2500 °C, while the color camera is limited to 1800 °C. The color camera works in the visible spectral range (400–700 nm) while the multispectral works in the near infrared (700–950 nm). In the latter, the intensity variation with temperature is lower, allowing a wider temperature range. Nonetheless, the color camera provides higher spatial resolution at a lower equipment cost and requires fewer processing steps than the multispectral one. The advent of extremely wide dynamic range color cameras for automotive could increase the temperature range while keeping the other advantages. An overall limitation of the color camera regards interference of ambient light, therefore low-temperature measurements (below 1000 °C) are possible only in a dark environment. The multispectral camera is more robust in this regard, since in lab or industrial environments near-infrared light is usually absent.

### 3.5. Results Comparison

**Figure 16:**
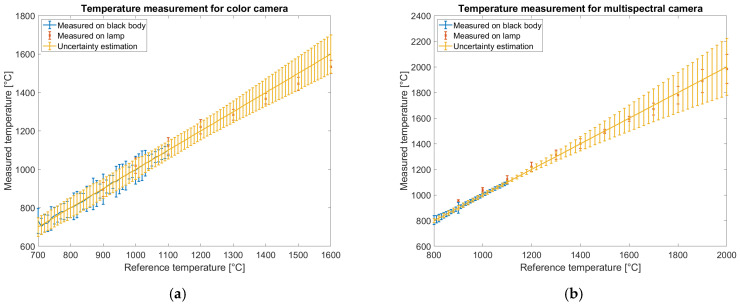
(**a**) Results comparison for the color camera. (**b**) Results comparison for the multispectral camera.

The overall comparison of temperature measurements using both the color and multispectral cameras is presented in Figure 16. Each plot shows measurements performed on the blackbody source and the lamp, along with the estimated Type B uncertainty, as described in Section 2.9. Blackbody images are acquired in the temperature range of 700 °C to 1100 °C for the color camera and from 800 °C to 1100 °C for the multispectral camera. The uncertainties for experimental data are estimated based on the standard deviation of the measured temperature within the region of interest. From the data shown in Figure 11b and Figure 12b, only the measurement corresponding to the optimized exposure time is reported. For the lamp, the data reflect the statistical uncertainty of the measurements.

Type B uncertainty accounts for the emissivity uncertainty (which has a negligible effect), the propagation of signal uncertainty via partial derivatives, and the uncertainty of the reference temperature, which increases outside the calibration range. All uncertainties are reported with a coverage factor of 2, corresponding to a 95% confidence level. A good agreement between the measured and predicted uncertainties is observed. If required by the application, the uncertainty range could be further reduced by averaging across more pixels. In this analysis, uncertainties are calculated assuming a 3 × 3 pixel region for the color camera and a 2 × 2 pixel region for the multispectral camera. The color camera exhibits higher uncertainty than the multispectral camera within the calibration range; however, its uncertainty increases more slowly with temperature, suggesting better suitability for extended-range temperature measurements.

### 3.6. Results of Alternative Approaches

**Figure 17:**
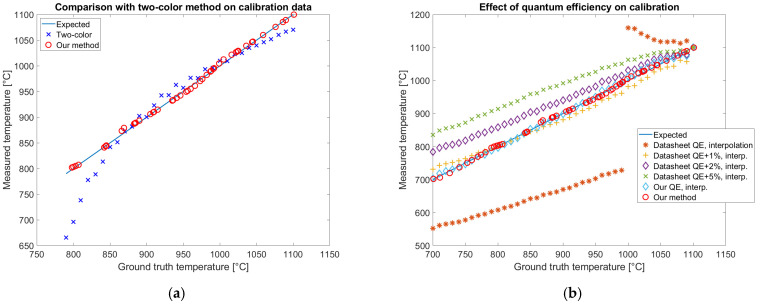
(**a**) Comparison of our results in calibration with the two-color method. (**b**) Comparison of our results in calibration with direct estimation of the temperature from the simulated Planckian locus with interpolations for several quantum efficiency datasets.

Alternative calibration approaches are the two-color method [17], presented in Section 2.10, and the direct estimation of the temperature from the simulated Planckian locus with temperature regression based on triangular interpolation [44], presented in Section 2.11. Both these methods have been tested on the calibration data of the color camera, after removing the dependence on exposure time. Results are reported in Figure 17.

For each method, the average, the standard deviation, and the root mean square of temperature prediction errors have been evaluated and reported in Table 4.

The results reported in Figure 17 and in Table 4 show that our method fits the calibration data better than all the alternative approaches that have been tested showing the lowest rms error of 3.7 °C. The temperature measured by the two-color method (Figure 17a) does not completely fit the variability of the data with a rms error of 35.5 °C. The best alternative approach to our method is the temperature measurement with the optimized QE and Ohno interpolation providing a rms error of 6.0 °C. The rms error is slightly higher than the one obtained with our method confirming that the neural network overperforms interpolation. If the datasheet QE is used with the same method, the temperature prediction error is almost two orders of magnitude worse. The modified datasheet QE allows the simulation of the data variability when a 0.02 constant is uniformly added, showing the necessity of an optimization step of QE in calibration.

## 4. Discussion

Our method proposes innovations over the state of the art regarding quantum efficiency estimation and temperature estimation from RGB color. These topics are discussed by comparing our results with previous ones in Section 4.1 and Section 4.2. In Section 4.3 the effect of exposure time is discussed. Limits of calibration are presented in Section 4.4.

### 4.1. Quantum Efficiency Estimation

The quantum efficiency estimation is a key step in camera calibration for temperature estimation. This result has been proven by simulating the signal collected by both a color and a multispectral camera for each band with the QE reported on the datasheet. The result does not match the experimentally observed gray levels on images acquired by looking at an Inconel 718 plate in a furnace at a known temperature. The discordance could be observed by comparing simulated and measured data reported in Figure 7b and Figure 8b. The relevance of QE on temperature estimation is shown in Figure 17b, where the datasheet QE is perturbed. On the other hand, ignoring the spectral overlap of the bands and approximating them as narrow, as done in the two-color method, introduces an error in the temperature estimation, as shown in Figure 17a. The temperature prediction by the two-color method shows a trend that could be attributed to the excessive simplification of the system. Figure 17b shows that the optimized QE allows for the estimation of the temperature, correctly capturing the complexity of the system both with our method based on a neural network and with an interpolation method. The adjustment performed by the optimization is less than 1% confirming the sensitivity of the color to temperature simulation to the uncertainty of QE. Our QE optimization method relies on images acquired by a furnace, approximating a black body source, typically available in labs where glowing hot parts are present. Alternative methods of inferring QE from several light-emitting diodes with different peak emission wavelengths [39] or using sets of narrow band filters [40] require dedicated tools and may not reach the required accuracy.

Limitations involve the convergence of the optimization method for QE that has been shown experimentally for both a color camera and a multispectral one, without providing full mathematical proof. Convergence happens since the spectra acquired at different temperatures are linearly independent, providing a robust dataset for the fit. Hyperparameters have been chosen by trial and error and a general rule to choose wavelength integration range, subsampling, weights, and weights decay cannot be formulated. Since the vast majority of the commercially available color cameras present similar datasheet QE, the hypothesis that the presented hyperparameters can guarantee general convergence appears reasonable. Moreover, the estimated QE does not differ so much from the datasheet one, therefore, it is believed to be reliable, even if it has not been verified with another technique. Finally, the estimated QE also considers the optical chain and the filters and does not directly provide information on the camera sensor itself.

### 4.2. Temperature Estimation from RGB Color

Our approach directly estimates temperature from RGB color, without converting the color space into a standard like CIE31 or CIE76. This choice presents the advantage of avoiding the uncertainty related to the conversion matrix. Furthermore, conversion is not possible or noisy when one of the bands shows a signal that is too dim or saturated. In this case, measuring in the color space of the camera potentially allows discarding the critical band and reconstructing the temperature from the remaining data. Conversion matrices, applied in the case of multispectral cameras to avoid band crosstalks [34,35], appears not applicable to color cameras since they do not capture the complexity of the QE of the system. Attempts to use a conversion matrix have been done in a temperature range above 1700 K [21], where limited temperature calibration references are available. For lower temperatures, the approach of modeling the Planckian locus in the color space of the camera relying on a black body source as a reference for calibration appears more robust. In our approach, temperature estimation from color is done by a neural network trained on the Planckian locus calculated after calibration. The results reported in Table 4 show that the neural network overcomes the interpolation method with a lower RMS on the measurement error. The network has been designed to generate a limited number of floating-point operations, that could be accelerated by dedicated hardware.

### 4.3. Effect of Exposure Time

The data reported in Figure 11, Figure 12, Figure 14 and Figure 15 show that the biggest source of uncertainty in temperature measurement is the effect of exposure time. The dynamic range of an 8-bit camera seems too limited to capture the variability of the intensity generated by glowing objects. In Figure 11 and Figure 12 a wide portion of the plot does not report the temperature measurement because the signal of at least one of the bands is too dim or saturated. In future works, a high dynamic range camera will be tested to improve robustness towards exposure time variation. Temperature measurement at different exposure times appears different, generating an uncertainty reported in Table 3 of 63.2 °C for the color camera and of 24.3 °C for the multispectral one. This uncertainty is attributed to the instability of the dark level, to the shot noise of the sensor, and to the quantization error. The first adds a bias to the signal, the second adds white noise on each reading and the last becomes relevant when exposure time is not optimal. The sources of uncertainty sum up influencing the data provided to the neural network and, therefore, the temperature estimation. A high dynamic range camera could reduce signal quantization uncertainty, potentially improving temperature measurement precision.

### 4.4. Limits of Calibration

The accuracy of the proposed calibration procedure depends on the combined spectral response of the imaging system and the radiative properties of the observed material. Any modification to the optical assembly that alters the system’s spectral transmission—such as replacing the lens with one made of different materials or coatings—requires recalibration. Similarly, changing the camera affects the quantum efficiency (QE) curve of the sensor, which directly influences the spectral response and also necessitates recalibration. The observed material is also a key factor. A change in the target material—such as switching to a different metal with a distinct emissivity slope—alters the spectral radiance profile and requires recalibration to preserve measurement accuracy.

Changes in working distance do not affect calibration unless they introduce significant atmospheric absorption, which could alter the spectral composition of the received signal. In typical laboratory or industrial conditions and over short distances, this effect is negligible, and recalibration is not required. However, in environments with long optical paths or significant temperature or gas composition gradients (e.g., high humidity or combustion gases), the spectral response could be affected, and recalibration may be necessary. Since this work is addressed to additive manufacturing that operates on short distances the effect of atmospheric absorption has been considered as negligible for future work.

In contrast, variations in aperture, focus, or exposure time do not influence the spectral response. In particular, the normalization procedure applied in this method effectively cancels the influence of exposure time, ensuring that such adjustments do not require recalibration. Overall, while recalibration is needed for any change that significantly affects spectral transmission or material emissivity, the procedure remains straightforward and can be conducted using standard tools available in a typical additive manufacturing laboratory.

## 5. Conclusions

Both an industrial color camera and a multispectral camera are used as thermal images of incandescent metals. A new calibration method is proposed, based on the estimation of the quantum efficiency of the sensor together with the optical chain including lenses and filters. The optimization algorithm compares the predicted observation with experimental data, acquired by looking at an Inconel 718 metallic substrate heated up in the temperature range from 700 °C to 1100 °C by a heat treatment furnace approximating a black body. To overcome the limited dynamic range of both cameras, a robust method for data preprocessing is proposed to keep into consideration images acquired at several exposure times. Images acquired at the same reference temperature with several exposure times are processed together obtaining a robust estimation of the light intensity collected in the different bands versus temperature, then an evolutive algorithm optimizes the quantum efficiency array of each band to simulate the experimental observations. The optimization method is tested with success both on an industrial color camera in the visible spectral range and on a multispectral camera in the near-infrared, refining the quantum efficiency of each band with respect to the one proposed in the datasheet. Refined quantum efficiency improves the correspondence of simulated radiation with measurements. Our method avoids the necessity of accurate quantum efficiency characterization by dedicated optical instrumentation, drastically reducing the cost of the thermal calibration of silicon CMOS cameras. The optimized quantum efficiency is used to generate a synthetic dataset in the temperature range from 500 °C to 3500 °C, corresponding to the Planckian locus relating the gray levels on all the bands to temperature in the camera color space. A neural network trained on the simulated synthetic dataset is used to regress temperature pixel by pixel. The network is tested on the calibration images showing a temperature measurement uncertainty, with a coverage factor of two corresponding to an interval of 95%, at 15.6 °C in calibration range for the multispectral camera and at 50.5 °C for the color camera. The results presented overperforms the two-color method that shows an uncertainty of around 70 °C, thanks to the full modeling of the quantum efficiency of each band. Furthermore, the uncertainty of our method could be reduced by averaging on multiple pixels. The multispectral camera provides better precision than the color camera. The generalization capability on the range from 1100 °C to 2000 °C is tested by looking at an incandescent filament bulb of a halogen lamp. The color camera provides 28 °C rms error on temperature measurement in the range of 900–1400 °C. The multispectral camera shows a rms temperature measurement error of 31 °C in the 1000–1400 °C range and 46 °C in the extended range from 1000 to 2600 °C. These generalization results are possible only in case of averaging on a wide Region of Interest since the pixel level measurement above 2000 °C appears subject to an uncertainty above 100 °C. Temperature measurement of the halogen bulb filament obtained by both cameras confirm the capability of the proposed calibration method to generalize at temperatures above the calibration range. The color camera has a reduced temperature operating range, but a higher spatial resolution than the multispectral one thanks to the reduced number of bands. The higher spatial sampling rate of the color camera helps in avoiding false reads generated by associating pixels sampled in regions with non-uniform temperature distribution. Both measurement accuracy and precision on calibration images remain strongly dependent on the exposure time of the image, opening the way for testing high dynamic range cameras in future work. Future work can also explore the option of hybrid training strategies of the neural network including both synthetic and real data or methods to strengthen the robustness of the network to noise.

## 6. Patents

No patents are reported in this manuscript.

## Figures and Tables

**Table 1:** Numerical hyperparameters for integration, dataset collection, and experimental setup.

Parameter	Color Camera	Multispectral Camera
Integration range	350–1100 nm	650–1050 nm
QE subsampling	10 nm	2 nm
Number of bands b	3 (red, green, and blue)	25 (from 700 to 950 nm)
Temperature range	1100 °C to 700 °C by 10 °C	1100 °C to 800 °C by 10 °C
Exposure time range	0.1 ms to 100 ms	10 ms to 316 ms
Optical filters	BP550 (visible pass)	ND200 (neutral density) and BP800 (near infrared pass)
Lens	LM50JC10M focal 50 mm	LM50JC10M focal 50 mm

**Table 2:** Contributions to the uncertainty for high levels of signal in both cameras.

Source of Uncertainty	Value	Distribution	Sensitivity Coefficient
Thermocouple	±4.4 °C	Rectangular	$\alpha_{T_{ref}}$
Color camera shot noise	2.78 DN	Poisson	$\frac{\partial f_{cal}}{\partial d_{b}}$
Multispectral camera shot noise	2.19 DN	Poisson	$\frac{\partial f_{cal}}{\partial d_{b}}$
$\mathrm{Emissivity}\;\mathrm{slope}\;\varepsilon_{1}=d\varepsilon /d\lambda$	σ $=0.1\;\mu m^{-1}\;$	Normal	$\frac{\partial f_{cal}}{\partial \varepsilon_{1}}$

**Table 3:** Results of network training.

Parameter	Color Camera	Multispectral Camera
Training error average (synthetic dataset)	−1.52 °C	−1.15 °C
Training error standard deviation (synthetic dataset)	3.10 °C	0.89 °C
Training error RMS (synthetic dataset)	3.45 °C	1.46 °C
Test error average (synthetic dataset)	−1.55 °C	−1.14 °C
Test error standard deviation (synthetic dataset)	3.30 °C	0.94 °C
Test error RMS (synthetic dataset)	3.64 °C	1.48 °C
RMS of estimation error (average on ROI)	6.32 °C	7.52 °C
RMS of standard deviation (pixel gray value)	63.21 °C	24.28 °C

**Table 4:** Results of alternative calibration methods. The minimum value of Rms is in bold.

Method	Mean Temperature Measurement Error	Standard Deviation of Prediction Error	Rms of Measurement Error
Our (700–1100 °C)	−0.9 °C	3.6 °C	**3.7 °C**
Two-color	−12.6 °C	33.8 °C	35.5 °C
Optimized QE, Ohno	1.3 °C	6.0 °C	6.0 °C
Datasheet QE, Ohno	−122.7 °C	183.7 °C	219.1 °C
Datasheet QE + 0.01, Ohno	−8.2 °C	17.4 °C	19.1 °C
Datasheet QE + 0.02, Ohno	42.0 °C	23.7 °C	48.1 °C
Datasheet QE + 0.05, Ohno	84.2 °C	38.6 °C	92.5 °C

## Data Availability

Dataset available on request from the authors.

## Abbreviations

The following abbreviations are used in this manuscript:

CMOS	Complementary Metal Oxide Semiconductor
RGB	Red Green Blue
QE	Quantum Efficiency
ROI	Region Of Interest
